# The analysis of F-actin structure of mesenchymal stem cells by quantification of fractal dimension

**DOI:** 10.1371/journal.pone.0260727

**Published:** 2021-11-30

**Authors:** Alla Revittser, Ivan Selin, Yuri Negulyaev, Vladislav Chubinskiy-Nadezhdin

**Affiliations:** 1 Group of Ionic Mechanisms of Cell Signaling, Institute of Cytology of the Russian Academy of Sciences, St-Petersburg, Russia; 2 Higher School of Software Engineering, Institute of Computer Science and Technology, Peter the Great St. Petersburg Polytechnic University, St. Petersburg, Russia; University of Virginia School of Medicine, UNITED STATES

## Abstract

The actin cytoskeleton is indispensable for the motility and migration of all types of cells; therefore, it plays a crucial role in the ability of the tissues to repair. Mesenchymal stem cells are intensively used in regenerative medicine, but usually relatively low percent of transplanted cells reaches the injury. To overcome this evident limitation, researchers try to enhance the motility and migration rate of the cells. As one of the approaches, co-cultivation and preconditioning of stem cells with biologically active compounds, which can cause actin cytoskeleton rearrangements followed by an increase of migratory properties of the cells, could be applied. The observed changes in F-actin structure induced by the compounds require quantitative estimation, and measurement of fluorescence intensity of the F-actin image captured by various microscopic techniques is commonly used nowadays. However, this approach could not always accurately detect the observed changes in the shape and structure of actin cytoskeleton. At this time, the image of F-actin has an irregular geometric pattern, and thus could be considered and characterized as a fractal object. To quantify the re-organization of cellular F-actin in terms of fractal geometry Minkovsky’s box-counting method is suitable, but it is not widely used nowadays. We modified and improved the previously described method for fractal dimension measurement, and successfully applied it for the quantification of the F-actin structures of human mesenchymal stem cells.

## Introduction

Fractals are irregular geometric patterns that are characterized by self-similarity and complexity. Traditional Euclidean geometry could not be applied to describe fractal objects as they have a non-integer value for their dimension, thus special fractal geometry is used to quantify the properties of the fractals [1]. Particularly, fractal dimension (FD) characterizes how fractal objects fill the space; the more space the object fills, the bigger is its FD. There are several fractal objects in cell biology, and fractal analysis was successfully applied as a quantification method to estimate the shape and morphology of neurons [2–4], membrane [5], cell boundaries [6], microtubules [7] and microfilaments [8].

The actin cytoskeleton is indispensable for the motility and migration of all types of cells; therefore, it plays a crucial role in the ability of the tissues to repair [9–11]. Stem cells are well known for their capabilities of differentiation and self-renewal, which allows these cells to fix damaged tissues. Transplantation of mesenchymal stem cells is a promising tool for regenerative medicine, but most stem cell-based therapies are currently at different stages of clinical research because of a number of various restrictions including the notion that relatively low percentage of cells successfully reaches the injured area [12, 13]. At this time, specific protocols such as co-cultivation and preconditioning of stem cells with biologically active compounds including those that influence microfilament organization, are being used as a potential approach to improve their regeneration properties [14, 15].

To quantify the re-organization of microfilaments F-actin is routinely stained with a fluorescent conjugate of selective F-actin binding phallotoxin phalloidin, or using with specific antibodies followed by visualization by fluorescence microscopy [16]. Commonly, to quantify the changes in F-actin structure, the average gray value of 2D image within the cell area (this is the sum of the gray values of all the pixels in the cell area divided by the number of these pixels), is used [17]. However, this approach that is based on the relative intensity of fluorescence could only provide the information whether less or more F-actin (that corresponds to actin disassembly or assembly, respectively) is in the cell after treatment with the reagents of interest. In more complicated cases, when the significant re-shaping or F-actin structure is observed, the approach becomes uninformative and could not be correctly used for statistically significant quantification of the changes in F-actin. Ten years ago, some scientific groups started to implement Minkovsky’s box-counting FD as a quantification tool for actin cytoskeleton structure [7, 18, 19], but, for some reason, this method had not become widely used. In this study, we modified and improved the previously described method for FD quantification of F-actin, and probed it as a tool for detection of the changes in F-actin structure of experimental model that are mesenchymal stem cells treated with F-actin disruptor latrunculin B [20].

## Materials and methods

### Cell culture and reagents

FRSN mesenchymal stem cells (dermal fibroblasts isolated from the foreskin of a 3-years-old boy) were obtained from the shared research facility “Vertebrate cell culture collection” supported by the Ministry of Science and Higher Education of the Russian Federation (Agreement №075-15-2021-683; the Institute of Cytology of the Russian Academy of Sciences, St. Petersburg) [21]. The cells were cultured in IMDM medium (Gibco, USA) supplemented with 10% FBS (Biowest, France) and antibiotics (penicillin 50 U/mL and streptomycin 50 μg/mL, Biolot, Russia). The seeding density was 10^4^ cells/cm^2^. The culture conditions were 37°C, 5% CO_2_, and 90% humidity. The culture medium was fully replaced every 2–3 days. Latrunculin B (Thermo Fisher, USA) stock solution was prepared by dissolving in DMSO according to the producer’s recommendations. The cells were plated on glass coverslips 2–3 days before the experiments. When cell monolayer achieved 50% density, coverslips were separated randomly into two control and five experimental groups. One control group was not treated with any reagents and another control group was incubated for 30 min in a culture media with 10 μM DMSO (that is the maximal concentration of the vehicle for the latrunculin B used in experiments). Five other groups were treated for 30 min with different concentrations of latrunculin B.

### F-actin staining and fluorescence microscopy

After the treatments, all samples were fixed in 3.7% paraformaldehyde for 10 min, permeabilized with 0.1% Triton X-100 for 7 min and stained with rhodamine conjugated to phalloidin (Rh-Ph, 2 μg/mL, Invitrogen, USA) for 30 min at 37° C. The nuclei counterstaining was performed with Hoechst 33342 (2 μg/mL, Sigma, USA) for 10 min. After each step, the samples were washed three times with PBS. Before the imaging, the samples were mounted on glass slides using Vectashield antifade reagent (Vector Labs, USA). Samples were visualized on Olympus FV 3000 laser scanning confocal microscope (Olympus Corporation, Japan) with a 40×/1.3NA oil objective. Lasers emitting at 561 nm or 460 nm and appropriate detectors for Rh-Ph or Hoechst 33342 were used to visualize F-actin or cell nuclei, respectively. The imaging parameters (laser power, detector gains and offsets) were kept constant between all the samples. To imitate the widefield fluorescence microscope we fully opened the pinhole (airy disk) of the laser scanning confocal microscope.

### Fractal dimension analysis

FD analysis was performed with the box-counting method that is a widely used algorithm for determining FD [1–8] of various fractal objects. To calculate the FD, an image of an object of interest is covered with a square grid (boxes, each box size is *q*), and then the number of boxes containing any part of the object inside them is calculated; the total number of boxes depends on the boxes’ size *N(q)*. During next step, the box size is reduced and calculation is performed again, all these steps is repeated several times to get *q→0*. The box-counting *FD* is: $FD=\underset{q\to 0}{\lim}\frac{\log N(q)}{\log1/q}$. The fractal object is approximated with a straight regression line if log (*q*) is plotted against log 1/*q*; hence the box-counting *FD* can be determined from the slope of this line.

**Fig 1:**
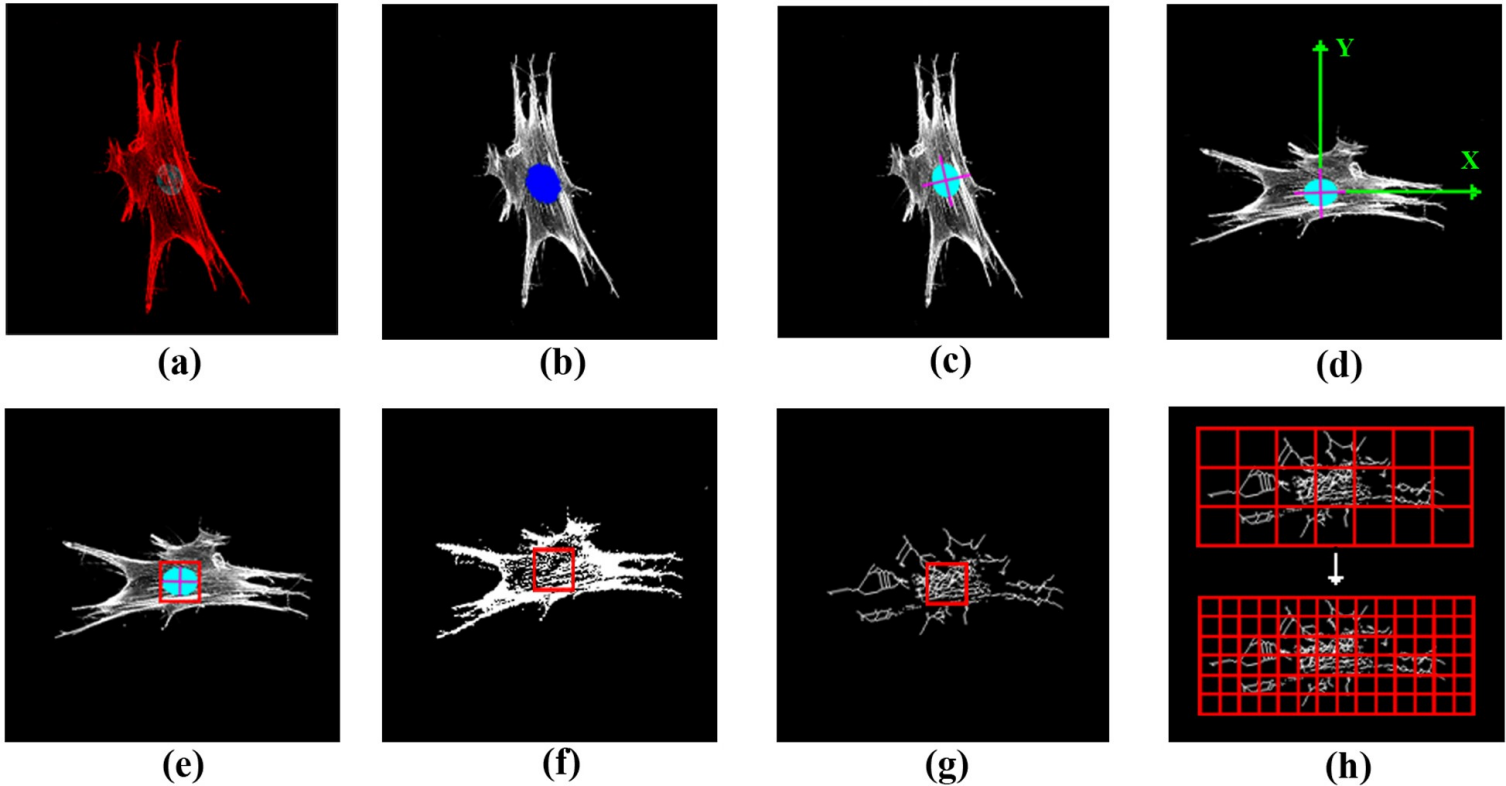
Step-by- step image processing algorithm for FD calculation. (a) The original two-channel image of the cell (F-actin and cell nucleus stained with Rh-Ph and Hoechst, respectively) was captured in 2048 x 2048-pixel resolution. (b) The blue channel (DAPI, cell nuclei) of the original image was isolated, binarized and color filled. (c) The minor and major axes of ellipse approximating the cell nuclei were found. (d) The original image is rotated in order that the major axis of ellipse becomes parallel to the X axis of the image. (e) The initial size of box grid is chosen as smallest square with power of two in which the ellipse approximating the cell nuclei is inscribed. The center of the box is in the ellipse’s centroid. The red channel (F-actin, Rh-Ph) of the original image is binarized (f), skeletonized (g) and processed using box counting method (h) using the calculated box size (e) in an initial iteration.

An analysis was performed using MATLAB (MathWorks, USA), data available on [22]. Before the analysis, each image of a single cell was separated manually and stored in color TIFF (Tagged Image File Format) of 2048 × 2048-pixel resolution (Fig 1A). To make the analysis invariant to picture size and rotation the image was aligned using the cell’s nuclei. The process is divided into 3 stages: (1) rotating the picture, (2) designing the box grid for the box-counting method and (3) FD calculation. The first stage consisted of several steps: firstly, the blue channel (cell nuclei) was extracted and binarized (Fig 1B). Then the biggest region (corresponded to nuclei) of at least 8-connected pixels was chosen. This region was approximated by an ellipse, and then the ellipses’ centroid, minor and major axes were calculated (Fig 1C). Next, the original image was rotated clockwise so that the major axis becomes parallel to the *X*-axis of the image (Fig 1D).

**Fig 2:**
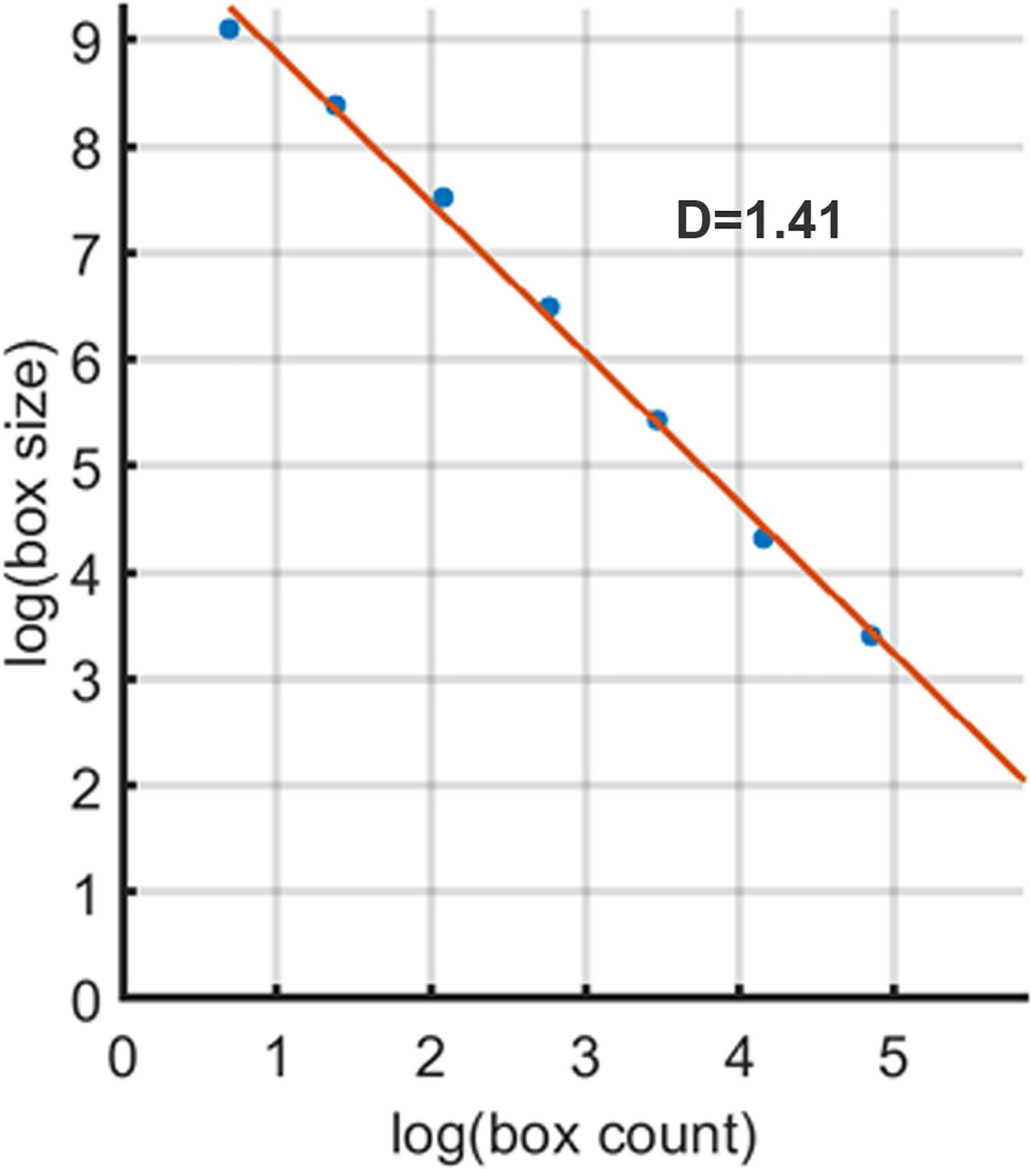
The representative result of FD measurement. FD value (D = 1.41) of the cytoskeleton of the cell presented on (Fig 1A) was defined as the slope of the line that approximates data points of plotting box sizes against box counts in logarithmic scale.

The second stage was aimed at the determination of the initial box grid parameters. Particularly, the box size was determined by getting the smallest square with a size of power of two, which could fit inside the nuclei’s approximated ellipse (Fig 1E). The box had an upper side parallel to the major axis, and the box center matched the ellipses’ centroid. For measurement of FD of the F-actin structures, the red channel (Rh-Ph) was extracted, binarized (Fig 1F) and skeletonized (Fig 1G). Then the obtained image was masked with a box grid of initial size both vertically and horizontally. After that, the number of boxes with any part of the structure inside was calculated (box count). Next, the box size was halved, and the number of boxes was calculated again. These steps were repeated until the size of the box side reached 2 pixels. FD of this image was defined as the slope of the line obtained by plotting log (box sizes) against log (box count, Fig 2). Finally, the skeletonized image was rotated around the centroid by 15° clockwise six times, and after each rotation, the FD value was calculated. The resulting FD was determined as the average value from seven measurements.

### Statistical analysis

Statistical analysis was performed using Matlab (MathWorks, USA). All data in groups were checked for normal distribution using Shapiro–Wilk test [23] for sample sets 5≤*n*≤50 or the Anderson-Darling test [24] for sample sets n>50. The data group is presented as mean *± S*. *D*. All datasets consisted of at least 15 measurements (*n =* 15). Statistical significance between the datasets was determined using Dunnett’s test [25] with an alpha value of 0.05 at a significance level lower than 0.05 (*p*< 0.05). To compare two different methods of fluorescent microscopy for capturing F-actin images, the Bland-Altman analysis was used (includes mean-difference plot and coefficient of determination R^2^) [26].

## Results

### Effect of latrunculin B on the F-actin structure of FRSN mesenchymal stem cells

**Fig 3:**
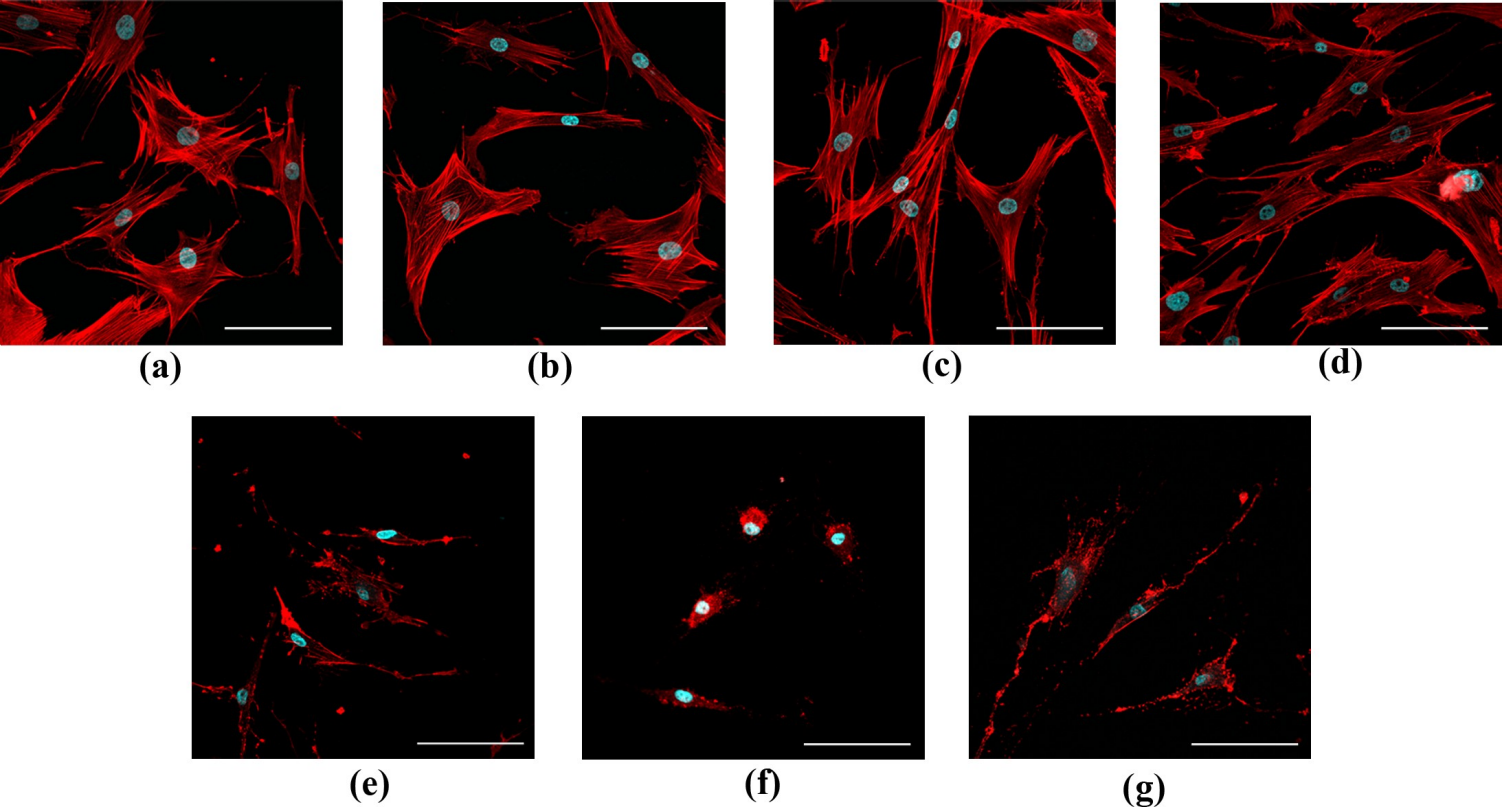
Representative images of F-actin staining (red channel) in FRSN cells in control and after 30 min incubation with Lat B. (a) Untreated cells and (b) cells treated with 10 μM DMSO (vehicle). (c-g). Cells treated with 10, 50, 500, 1000 and 10000 nM Lat B, respectively. Cell nuclei were counterstained with Hoechst (blue channel). Scale bar is 100 μm.

To probe whether the calculation of FD could detect the changes in F-actin reorganization in MSCs we had performed the experiments on FRSN cells treated with latrunculin B (Lat B), a biologically active agent that blocks the polymerization of actin monomers (Fig 3). One control group was not treated with the reagent (Fig 3A), while the other control group was incubated for 30 min in a culture media with 10 μM DMSO; that is maximum concentration as a vehicle for Lat B (Fig 3B). Five other groups were incubated for 30 min in a culture media with 10, 50, 500, 1000 nM and 10 μM of Lat B, respectively (Fig 3C–3G). No visible changes in cellular F-actin between control cells and cells that were incubated with 10 nM and 50 nM of Lat B were observed. At the same time, a noticeable perturbation in F-actin organization in the cells which had been incubated with 500, 1000 nM and 10 μM of Lat B could clearly be seen. To quantify the effect of Lat B on F-actin structure in terms of FD we applied the protocol described in the Methods section.

### Distribution of fractal dimension values for F-actin structures of a random cell

**Fig 4:**
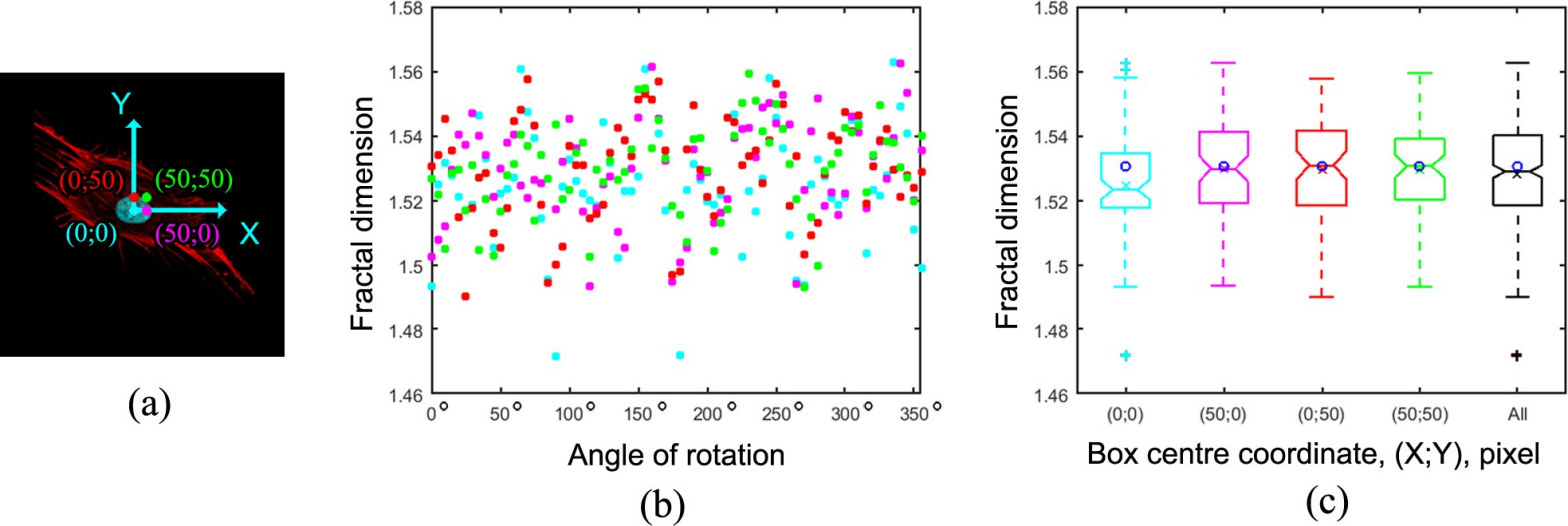
Distribution of fractal dimension values for F-actin image of a random cell on different angles and box positions. (a) Representative F-actin image of the cell from control group. Color points and pixel coordinates (cyan, green, red, and magenta) shows the positions of 4 box centers during box counting process. (b) dot plot shows variation of FD values which were obtained during the variation of the positioning of box centers and rotation angles. (c) notched boxplots of FD values (averaged for values obtained by varied positioning of box center and rotation angles). Black boxplot represents the cumulative FD values for all angles and box positions. Mean values are shown as color-coded crosses (x) on the boxplots. FD value (calculated using the previously described algorithm, see Methods) is shown as blue circles for comparison of the value with the medians and mean values of current FD distributions.

Firstly, we performed the calculation of FD of the F-actin structure of the random cell from the untreated control group. Initially, we applied our original algorithm (without averaging step) that is based on the placement of the center of the box of determined size (see Sec. 2.3) in different locations followed by FD calculation at every 5° during the 360° rotation of the image (Fig 4). The color points on Fig 4A show four positions of the center of the boxes used for calculation: the cyan point shows the position of the center of an ellipse that circumscribes the nuclei, and the red, magenta and green points are shifted by 50 pixels in indicated directions (Fig 4A) from the position of the cyan point. Fig 4B shows the dependence of FD values (Y-axis) calculated for different box positions (color-coded in Fig 4A) from the degree of image rotation (X-axis). The obtained FD values varied in the range of 1.47–1.56 (for 284 different angles and box positions).

Next, we checked if the mean FD value calculated for the dataset obtained from the image during rotation around one of four previously chosen positions (Fig 4A) reflects the exact value of FD of the F-actin structure of the cell. Particularly, we plotted four notched colored boxplots (Fig 4C, cyan, magenta, red and green, n = 71 for each group) and black boxplot which represents the cumulative FD values for different angles and box positions (see Fig 4C, n = 284). The presentation of the data as a notched boxplot allows to visualize several important statistical parameters including upper and lower values, outliers, 25th and 75th percentiles, median and 95% confidence interval of median of the distribution. In addition, we added mean values as color-coded crosses (x) on the boxplots, and the FD value (shown as blue circles inside the notched boxplots) we had calculated using the previously described algorithm (see Methods) to compare it with medians and mean values of current FD distributions Fig 4C. Importantly, we observed that both calculations resulted in close results (the difference between them is less than 0.01). Also, the plots indicated that these distributions are close to normal (close values of their mean and median, as well as symmetry and shape of distributions). Importantly, the Anderson–Darling test confirmed that the data (Fig 4C, black boxplot, *n* = 284) is distributed normally, thus it could be characterized by its mean value. As we observed previously, the median and the mean value of distribution (Fig 4C, black boxplot, n = 284) was close to the FD value which was calculated by our algorithm, so we concluded that our approach could be applicable for measuring of FD for the F-actin structures of the cells.

### FD analysis of morphological changes in cellular F-actin after treatment with latrunculin B using confocal and widefield fluorescent microscopy

**Fig 5:**
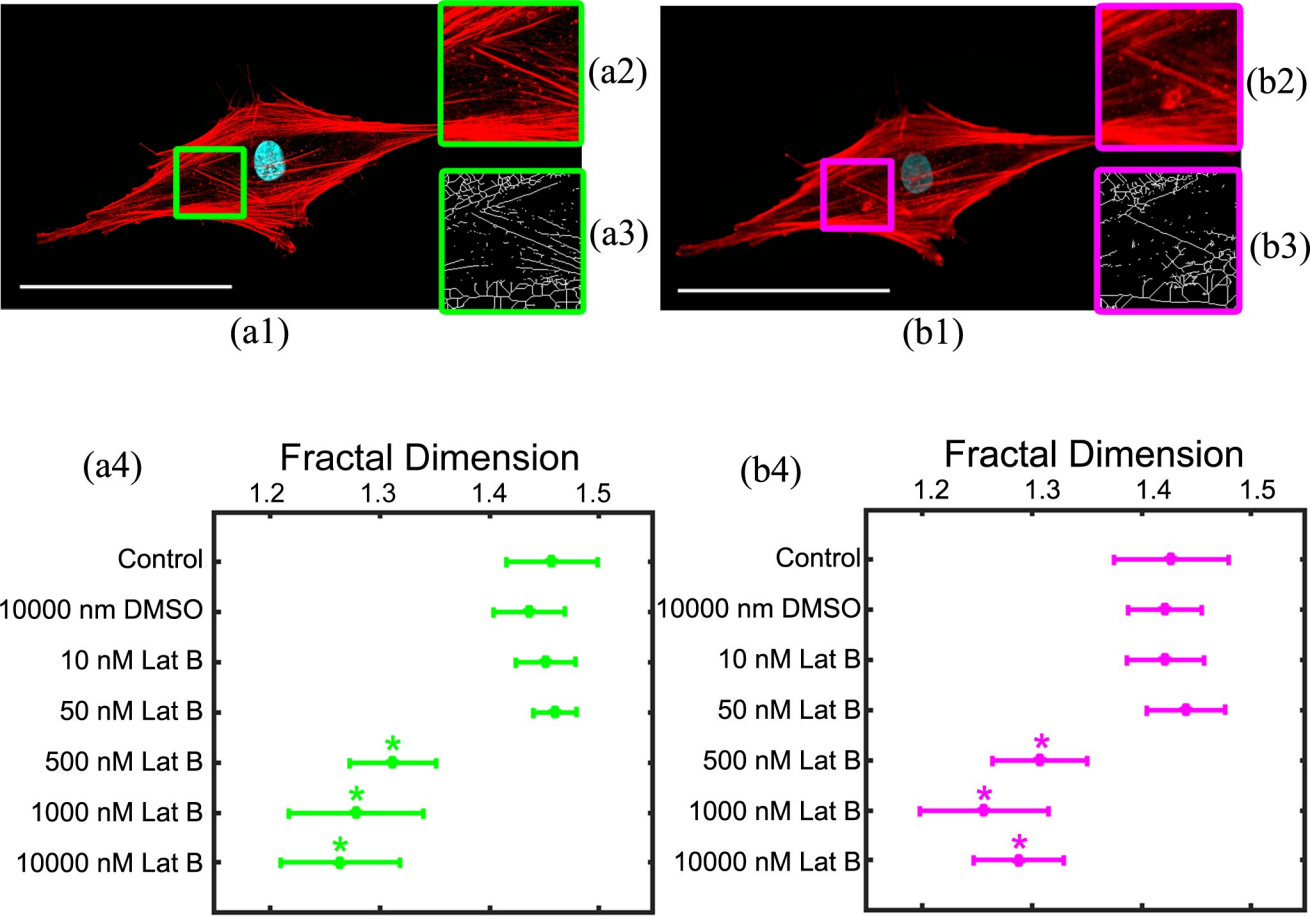
FD analysis of F-actin changes after treatment with Lat B using confocal or imitating widefield fluorescent microscopy. Images of random cell from the control group were obtained by confocal (a1) or widefield fluorescent (b1) microscopy. Scale bar size is 100 μm, (a2) and (b2) show double-scaled images, and (a3) and (b3) show its’ skeletonized versions. (a4) and (b4) represent FD mean values and their standard deviations calculated for groups of control and Lat B-treated cells (n = 15 cells in each group) where F-actin images were obtained by confocal or imitating widefield fluorescent microscopy respectively. (*) comparing to DMSO-treated control group, p<0.05, each group is n = 15 cells.

Using our algorithm, we measured FD of cellular F-actin after treatment with Lat B (Fig 5). Further, we analyzed the images acquired by confocal microscopy or by imitation of widefield fluorescent microscopy (CFM, FD values in brackets) to probe if our approach is sensitive to the microscopic technique used for acquiring the F-actin images.

**Fig 6:**
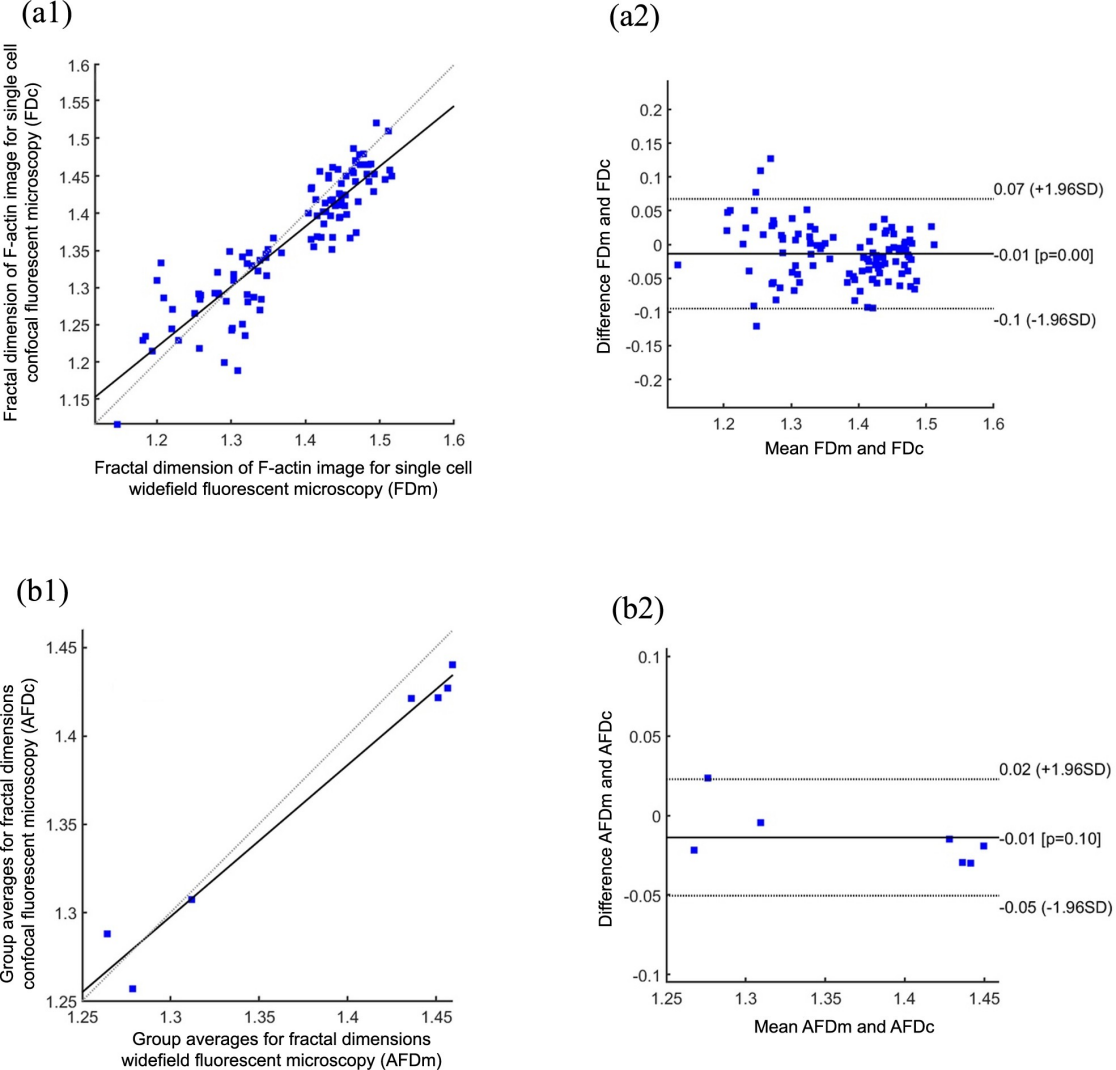
Correlation plot and Bland-Altman plot for FD values calculated for F-actin images captured using confocal and widefield microscopy techniques. (a1) and (a2) for FDs of all values which had been used in analysis (n = 105). (b1) and (b2) for average values of groups (n = 7) which were treated with different concentrations of Lat B (n = 15 cells in each experimental group).

Particularly, for the control group and groups treated with 10 μM DMSO, 10 nM or 50 nM Lat B FDs were: 1.46±0.04 (CFM = 1.43±0.05), 1.44±0.03 (1.42±0.03), 1.45±0.03 (1.42±0.04), 1.45±0.02 (1.44±0.04) respectively (no significant difference from the control group was detected using Dunnett’s test, p>0.05). FD values for groups treated with 500 nM, 1000 nM and 10 μM Lat B were 1.31±0.04 (CFM = 1.31±0.04), 1.28±0.06 (1.26±0.06) and 1.26±0.05 (1.29±0.04) respectively, and they were significantly different from the control group (Dunnett’s test, p<0.05). In sum, we obtained close FD values from analysis of confocal and widefield microscope images, and we compared them using the correlation plot and Bland-Altman (mean-difference) plot (Fig 6).

For F-actin structures of all cells (n = 105) measured by our algorithm, the coefficient of determination was R^2^ = 0.80 (P<0.05%, sum of squared errors SSE = 0.15) which means a high correlation between FD values measured from confocal and widefield microscope images (Fig 6A1); similar situation was for average values of FDs (Fig 6B1), R^2^ = 0.97, P<0.05%, SSE<0.01. The Bland-Altman plot (Fig 6A2) showed that 97% of dots were in agreement interval, mean was -0.01 and bias was 0.08, these values were small compared to FD values; a similar situation was observed for average values of FD (Fig 6B2), 98.4% of dots were in agreement interval, mean 0.01 and bias 0.03. These numbers indicated that the calculation of FD for F-actin images obtained by different imaging techniques (confocal or widefield fluorescence microscopy) led to the same results.

## Discussion

The FD measurement was shown to be as a useful tool to estimate the organization of cellular F-actin in several studies [8, 14]. However, this method has not become widely used in biological research, although the number of publications that mention FD is growing. Our study presents an improved approach to quantify the changes in cellular F-actin organization by calculation of FD that is based on automatically chosen parameters as box sizes, positions and averaging algorithms. We used cultured FRSN stem cell line as an experimental object that is characterized with developed actin cytoskeleton and prominent stress fibers. Our method allowed us to describe the differences in F-actin organization between control cells and cells treated with an inhibitor of actin polymerization Lat B in terms of FD. We observed the decrease of FD after latrunculin treatment and it correlates with the fact the FD is related to complexity of the object: Minkovsky’s FD of the image is a measure of how objects of interest fill the available space, thus the higher the value of FD is, the more space is filled by the object. The decrease of FD indicates that amorphous actin structures observed after incubation of the cells with Lat B fills less space than distinct developed actin stress fibers in control cells (Fig 3). The significant changes of actin structures caused by Lat B in FRSN cells was expected based on well-documented mechanism of inhibition of actin polymerization [27].

We discovered that the FD value of the actin cytoskeleton depended on the image rotation angle and box grid position. Thus, the FD value could significantly vary while capturing the image of the cell that is positioned randomly, as well as using a random position of the box grid. Therefore, a single value calculated from a randomly positioned cell could not appropriately reflect a real FD value of F-actin in this cell. To overcome the problem, we tested if the average FD value calculated from a whole dataset of FD values obtained on different cell and box grid positions reflects the real FD value for the F-actin structure. We found that the calculated dataset of FD values for a random single cell was characterized by a normal (Gaussian) distribution. Thus, the average FD value could be taken for further calculations and comparison of FD value of F-actin between the different experimental conditions. A similar problem of FD value calculation on different angles of image rotation was previously discussed for F-actin of neurons [3], and the problem was solved by the same way using the image rotation and averaging of resulting values.

Also, for a correct calculation of FD values it is important to correctly choose the range of sizes where the log(box size) vs log(box count) is approximated by the linear dependence. The actin cytoskeleton exists on a limited size scale that is practically between the size of the whole cell itself (up to 150 μm for several cell types) and the size of single globular actin [27] (about 4 nm). Most often, the biggest box size is defined manually, for example, researchers randomly chose 207 pixels for the confocal image of cardiac fibroblasts’ actin cytoskeleton captured using 40× objective [8]. In our approach, we were able to automate the process of box size definition by suggesting that the size of cell nuclei could be used as reference points for the biggest box size. To simplify further processing we took not the precise size of the cell nucleus but the closest value (in pixels) that was the bigger power of two (maximal box size for the first iteration of FD calculation is ≈2^n^ pixels of the real size of the nucleus). Then, during the calculation, we reduced the box size by 2 (reduced size of box for the second iteration is 2^n^/2) each time during the calculation until the final box size was 2 pixels. For an in vitro growing (during the interphase) of fibroblast-like cells (including stem cells) in a 2D environment the nuclei area is smaller than 40% of the total cell area but is still big enough for choosing several points for log(box size) vs log(box count) graph. Notably, our improved technique for the FD calculation is applicable for quantification of FD of actin cytoskeleton of any fibroblast-like cells that meets the criteria of the nuclei/total cell size ratio described above.

In our experiments, we utilized the 40× objective to capture the images, thus the size of the biggest box was about 20 μm (128 pixels, 128 = 2^7^) while the size of a smaller box was about 311 nm (2 pixels, 2^1^ = 2) for all 100 cells used for calculation. Thus, we had n = 7 points for the log (box size) vs log (box count) graph. Importantly, we gained almost linear dependence (R^2^ coefficient was between 0.999–0.991) at the selected scale so a nuclei size can be successfully used for the determination of the sizes of box grids. Also, it should be taken into account that the minimal box size must be larger than the limit of resolution for a confocal microscope (that is near 200 nm).

We performed our modified fractal analysis for FD calculation of F-actin in FRSN cells, and to obtain diverse data sets for the analysis we had two control groups (untreated cells and cells incubated with 10 μM DMSO as a vehicle for latrunculin B), and five groups treated with different concentrations of latrunculin B (from 10 nM up to 10 μM). We confirmed the statistical differences between control and treated experimental groups, and these results highly correlated with visual evaluation of actin structures in the images. Also, we obtained two types of images for each sample set that were captured by (1) confocal microscopy and (2) by the imitation of a widefield fluorescence microscope. The FD analysis of both image types showed similar results confirmed by Bland-Altman graphs. Thus, our approach to fractal analysis is applicable for the evaluation of F-actin organization in stem cells (even for small datasets, 15 single cells in each experimental group). Most importantly, our analysis could be used for the F-actin images captured using a simple widefield fluorescence microscope, which eliminates the requirement for technical equipment of the laboratory with expensive high-resolution microscopic systems.
